# Comparative Study on the Functional Connectivity of Amygdala and Hippocampal Neural Circuits in Patients With First-Episode Schizophrenia and Other High-Risk Populations

**DOI:** 10.3389/fpsyt.2021.627198

**Published:** 2021-09-01

**Authors:** Xinrui Wang, Zhiyang Yin, Qikun Sun, Xiaowei Jiang, Li Chao, Xu Dai, Yanqing Tang

**Affiliations:** ^1^Department of Psychiatry, The First Affiliated Hospital of China Medical University, Shenyang, China; ^2^Brain Function Research Section, The First Affiliated Hospital of China Medical University, Shenyang, China; ^3^Department of Radiotherapy, The First Affiliated Hospital of China Medical University, Shenyang, China; ^4^Department of Radiology, The First Affiliated Hospital of China Medical University, Shenyang, China; ^5^Department of Geriatric Medicine, The First Affiliated Hospital of China Medical University, Shenyang, China

**Keywords:** schizophrenia, functional connectivity, amygdala, hippocampus, high risk

## Abstract

**Objective:** Cortical-limbic system neural circuit abnormalities are closely related to the onset of schizophrenia (SZ). The amygdala, hippocampus, cingulate, and prefrontal lobe are important components of the loop. In this study, we compared resting-state functional connectivity (rs-FC) between the amygdala/hippocampus and cingulate/prefrontal regions among patients with first-episode schizophrenia (FE-SZ), high risk populations with SZ (HR-SZ), and healthy controls (HCs). By discovering the abnormal pattern of the cortical-limbic system of SZ and HR-SZ, we attempted to elucidate the pathophysiological mechanism of SZ.

**Method:** This study collected seventy-five FE-SZ patients, 59 HR-SZ, and 64 HCs. Analysis of variance and chi-square tests were used to analyze their demographic data. Analysis of covariance and *post-hoc* analysis were performed on the functional connectivity of the three groups. Finally, correlation analysis between the significant brain functional connectivity value and the scale score was performed.

**Results:** The results of the analysis of covariance showed that there were significant differences in rs-FC between the amygdala and the right middle cingulate and between the hippocampus and the bilateral medial superior frontal gyrus among the three groups (Gaussian random field (GRF)-corrected voxel *p* < 0.001, cluster *p* < 0.05). *Post hoc* comparisons showed that the rs-FC of the amygdala—right middle cingulate and the hippocampus—bilateral medial superior frontal gyrus in patients with SZ was significantly lower than that of HR-SZ and HC (Bonferroni corrected *p* < 0.001). There was no significant difference between the HR-SZ and HC groups. The results of the correlation analysis showed that rs-FC of the hippocampus-medial frontal gyrus in patients with SZ was positively correlated with core depression factor scores on the Hamilton Depression Scale (*P* = 0.006, *R* = 0.357).

**Conclusion:** There were different patterns of functional connectivity impairment in the amygdala and hippocampal neural circuits in the schizophrenic cortical-limbic system, and these patterns may be more useful than genetics as state-related imaging changes of the disease.

## Introduction

Schizophrenia (SZ) is a severe mental disorder whose etiology and pathogenesis have not been elucidated to date. SZ is characterized by uncoordinated mental activity and responses to the environment and it affects many aspects of mental activity, such as thinking, perception, cognition, emotion, and behavior. SZ disorders (1) are accompanied by abnormalities in brain structure and function (2, 3). The lifetime prevalence of SZ in the general population is ~0.5% (4, 5).

SZ is a highly hereditary mental disorder. Relatives of patients with SZ share the risk genes of schizophrenia and are at a higher risk of manifesting the disease (6). Family studies have shown that the prevalence of SZ among first-degree relatives of SZ is ~6–17%. For the children of people with SZ, if one of their parents has it, their risk is ~13%. If both parents have SZ, the risk can reach 46% (7, 8). Therefore, the first-degree relatives of patients with SZ belong to a population with a high risk of SZ. Among them, the children of SZ patients are considered to be at a genetically high risk of SZ (HR-SZ). The study of HR-SZ helps to understand the triggers and underlying mechanism of SZ without the confounding effects of symptoms. Biomarkers shared by people with SZ and HR-SZ but not HCs may be genetic and psychosis indicators. Biomarkers that distinguish HR-SZ from SZ patients and HCs may be protective indicators against the penetrance of schizophrenia genes.

A popular hypothesis in schizophrenia research is that schizophrenia is a brain “connectivity imbalance” disease whose symptoms are thought to be due to abnormal interactions between different brain regions (9). Magnetic resonance imaging has become the main research method to verify this hypothesis. Among these studies, structural and functional impairment of the SZ cortex-limbic system is one of the most commonly reported findings. The core areas of this neural circuit are the amygdala, hippocampus, cingulate cortex, and prefrontal cortex (PFC) (10). Studies of healthy individuals have shown that emotional processing is mediated through the interaction of the ventral and dorsal systems of the cortical-limbic circuit, with the ventral system centered on the marginal zone, especially the amygdala, which participates in emotional information assessment, and the subsequent dorsal system, including the dorsal region of the prefrontal cortex (PFC) and the anterior cingulate cortex (ACC), which can regulate emotional responses (11, 12). Among patients with SZ, a study reported that compared with HCs, abnormal expression and regulation of emotion in patients with SZ are associated with the amygdala and anterior cingulate cortex (ACC) and dorsal lateral prefrontal cortex (DLPFC) (10). Studies have also reported that the connectivity variability between the amygdala and the medial prefrontal cortex (mPFC) in patients with SZ was increased (13). The hippocampus, as an important part of the limbic system structure, involves learning, memory, analysis and integration of information and is also related to the occurrence of emotions such as anxiety and fear (14). Disturbance of the interaction between the PFC and hippocampus is thought to be the cause of cognitive deficits related to working memory in patients with SZ (15). However, Meyer Lindenberg and colleagues reported that the functional connectivity between the right DLPFC and the left hippocampus was not reduced in patients with schizophrenia under a working memory task test (16).

Based on previous studies, we can speculate that there may be different cortical-limbic abnormalities in SZ, but the number of studies is limited, and the results are contradictory. Previous studies also found that compared with patients with SZ, unaffected first-degree relatives of patients with SZ may show mild neuroimaging abnormalities (17–19). There is good evidence for subtle abnormalities of hippocampal volume in relatives that are not as marked as the deficits in overt schizophrenia. In addition, the functional imaging literature suggests that prefrontal cortex function may deteriorate in those at risk who go on to develop the disorder (20). Twins study found the more an affected twin of SZ differed from the unaffected twin in left hippocampal volume, the greater they differed in prefrontal physiological activation during the Wisconsin Card Sorting Test. In the affected twins as a group, prefrontal activation was strongly related to both left and right hippocampal volume. However, these relationships were not found in the group of unaffected twins (15). A combined twin and family design examined four prefrontal cortical regions of interest. Superior and inferior regions were significantly smaller in SZ patients; however, the volumes of these same regions were normal in unaffected relatives, while in contrast, prefrontal (executive) functioning deficits were present in the unaffected relatives (21). Studies related to the cortico-limbic system have reported that young offspring of people with SZ are at risk for schizophrenia and that the amygdala and hippocampus are reduced in volume compared to healthy people (22, 23). Functional studies have also found that in the offspring of SZ, the functional connection between the amygdala and the brainstem is increased (24). Although changes in the cortical limbic system have been found in studies of schizophrenia and its relatives, the results were in-consistent, research on its function in SZ genetic relatives is relatively limited especially in the offspring of SZ, which needs further research.

In this study, we used functional magnetic resonance imaging (fMRI) to study the changes in functional activity in the cortical-limbic system between the amygdala/hippocampus and prefrontal lobe/cingulate by comparing the first episode of SZ (FE-SZ) and HR-SZ (offspring of SZ) in the resting state. We attempted to explore the patterns of schizophrenic cortical-limbic system impairment and genetic imaging changes of disease on the offspring of SZ, and further elaborate on the pathophysiological mechanism of SZ.

## Methods and Materials

### Design, Participants, and Procedure

A total of 198 subjects were collected in this study. The age range for subjects recruited in this study was 13–35 years. Seventy-five FE-SZ patients (48 females and 27 males) with a course of <24 months were recruited from the Department of Psychiatry of the First Affiliated Hospital of China Medical University and Shenyang Mental Health Center. At least two psychiatrists had made the same diagnosis, consistent with the fourth edition of the Diagnostic and Statistical Manual of Mental Disorders (DSM-IV) for schizophrenia or schizophreniform conditions. Structured Clinical Interview (SCID) assessments were applied to patients aged 18 years or over. Patients under 18 years old were assessed using the Kiddie Schedule for Affective Disorders and Schizophrenia for School Age Children, Present and Lifetime Episode (K-SADS-PL). Patients did not combine other mood disorders. Fifty-nine HR-SZ subjects (25 females and 34 males) were included. At least one of their parents met the diagnostic criteria for SZ in DSM-IV. No mental illness was diagnosed in the HR-SZ subjects. Sixty-four HC subjects (33 females and 31 males) were recruited as healthy control groups. None of the individuals in the control group had been diagnosed with DSM-IV-related diseases and they had no family history of mental illness. The Brief Psychiatric Rating Scale (BPRS), Hamilton Depression Scale (HAMD) and Hamilton Anxiety Scale (HAMA) were used to evaluate their clinical symptoms.

Exclusion criteria for the participants: (1) A history of a major medical disease, such as diabetes and hypertension. (2) Neurological diseases, such as cerebrovascular disease, epilepsy, brain tumors, etc. (3) A history of severe head trauma (loss of consciousness lasting more than 5 min); (4) Neurodevelopmental disorders; (5) Thyroid diseases, multiple sclerosis and diseases that can cause mood disorders; (6) Substance abuse or dependence; (7) Those who must use medications that affect the central nervous system or the mood; (8) Contraindications to MRI; (9) Pregnant women. Anyone who met the exclusion criteria was excluded from the study.

This study was approved by the Ethics Committee of China Medical University. All participants were voluntarily participating. All participants were informed of the experimental content in detail before the experiment and could withdraw at any time. Informed consent was signed by them or their guardian.

### MRI Data Collection and Processing

The MRI scan of this study was completed by a trained radiologist. The scan was conducted in the Imaging Department of the First Affiliated Hospital of China Medical University. The scanning process was attended by the evaluators. The MRI used a 3.0 T superconducting scanner, produced by General Electric (Milwaukee, USA). The software version used was Release 8.3. It was equipped with planar echo imaging software and hardware equipment. The standard head coil facilitates signal reception. Before the scan, the subjects were told to close their eyes, keep their bodies still, try not to think and to not fall asleep during the scan. Wearing sound-proof earplugs reduces the impact on the subject of the noise emitted by the MRI during the scan.

### MRI Data Processing

#### Data Preprocessing

The DPABI software package (a toolbox for Data Processing & Analysis for Brain Imaging) (25) was used to preprocess the magnetic resonance raw data. This software package runs on the MATLAB software platform and is widely used in the field of fMRI research. The steps are as follows: convert the original DICOM format data into computer processable format data (.nii format); remove the first 10 time points; unify the images acquired at different times to a time point through reasonable transformation to reduce the difference; head movement correction (if the head moves too much, the data should be excluded from the analysis. In this study, the head translation of all subjects was <3 mm, and the head rotation was <3° after spatial standardization); used a sampling resolution of 3 × 3 × 3 mm to normalize the functional magnetic resonance images of all subjects to the Montreal Neurological Institute (MNI) standard space; smoothed it with 6 mm × 6 × 6 mm; remove linear drift; reserved the band of 0.01–0.08 Hz to remove high-frequency physiological noise; and regression of the interference of the signaling variables (included white matter signal, cerebrospinal fluid signal and global signal). Four HC subjects, 5 HR-SZ subjects and 12 FE-SZ subjects were excluded.

#### Region of Interest (ROI) and Template Production

According to the anatomical automatic labeling (AAL) template included in DPABI, the ROIs of the bilateral amygdala and bilateral hippocampus were defined and resampled to 3 × 3 × 3 mm^3^. For each subject, the time series of voxels in the ROI were averaged to generate the average time series of the ROI. According to the AAL template, a template for the prefrontal and cingulate cortex was made. Differences between groups in the secondary template area were compared when performing group-to-group statistics.

#### Calculation of Functional Connection (FC)

For each subject, the DPABI software package was used to calculate the correlation between the average time series of the two ROIs and the time series of each voxel in the gray matter (except the cerebellum) region. Then, the Fisher r-to-z transform was used to convert the correlation coefficients into functionally connected z-values, that is, FC values.

### Statistical Analysis

Using SPSS 22.0 (Statistical Product and Service Solutions) statistical software, the clinical data of the three groups of subjects were statistically analyzed by chi-square test and analysis of variance, and the statistical methods of one-way ANOVA and chi-square test were used. The DPABI software package was used to perform an analysis of covariance (ANCOVA) on the FC results of the three groups of subjects, and Gaussian random field (GRF) correction was performed. The correction threshold with voxel *p* < 0.001 and cluster *p* < 0.05 was considered statistically significant. A *post-hoc* analysis was performed afterward, and Bonferroni correction was used with *p* < 0.05 considered to indicate statistical significance.

The rs-FC values of the brain regions with significant differences during the comparison were extracted and correlated with the scores of the clinical scales BPRS, HAMD, and HAMA.

## Results

### Demographic and Clinical Scales

A total of 198 subjects were included in this study, including 75 FE-SZ, 59 HR-SZ, and 64 HC. There were statistically significant differences between the three groups in terms of age (*F* test, *F* = 10.285, *p* < 0.001) and gender (chi-square test, *df* =2, x^2^ = 6.828, *p* = 0.033). Therefore, age and gender were used as statistical covariates (the results were shown in Table 1).

**Table 1:** Statistical results of the clinical data.

	**HC**	**HR-SZ**	**FE-SZ**	***F*/χ^**2**^**	**P**
	***n* = 64**	***n* = 59**	***n* = 75**		
Gender (Male/Female)	33/31	34/25	27/48	6.828	0.033^*^
Age (year)(Mean ± SD)	23.016 ± 6.11	21 ± 5.15	18.947 ± 4.57	10.285	<0.001^**^
BPRS total score(Mean ± SD)	/	/	36.959 ± 12.77	/	/
HAMD total score(Mean ± SD)	/	/	8.596 ± 7.91	/	/
HAMA total score(Mean ± SD)	/	/	7.173 ± 7.23	/	/

* *p < 0.05;*

** *P < 0.001*.

*Multiple comparison correction (age): HC vs. HR-SZ P = 0.141, HR-SZ vs. FE-SZ P = 0.052, HC vs. FE-SZ P < 0.001*.

### Amygdala rs-FC Results

**Figure 1:**
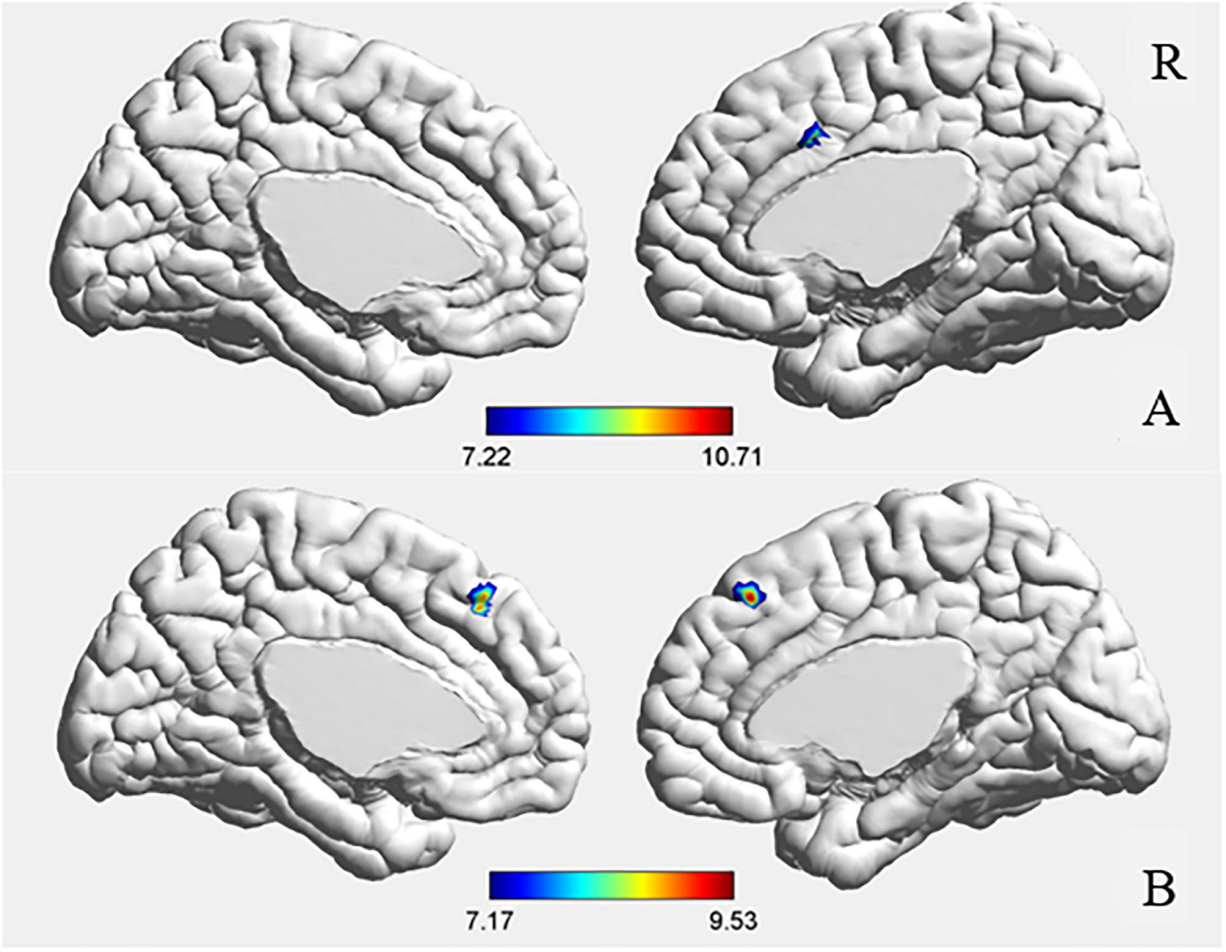
ANCOVA results of amygdala **(A)** and hippocampus **(B)** functional connectivity. GRF corrected, voxel *p* < 0.001, cluster *p* < 0.05.

**Figure 2:**
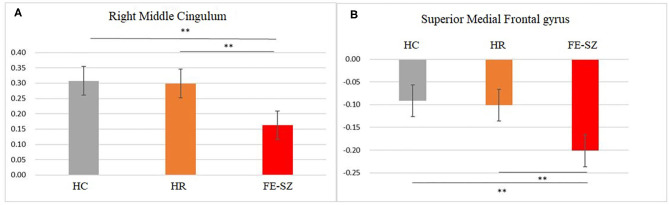
*Post hoc* results of amygdala **(A)** and hippocampus **(B)** functional connectivity. **Bonferroni corrected, *p* < 0.001.

The ANCOVA results showed that only the right middle cingulate had significant differences after correction (GRF correction, voxel *p* < 0.001, cluster *p* < 0.05, coordinate showed in Table 2). *Post hoc* analysis showed that compared with the other two groups, FE-SZ had a lower amygdala-right middle cingulate rs-FC (Bonferroni correction *p* < 0.05, also *p* < 0.001), and there was no significant difference between HR-SZ and HC (*p* > 0.05) (the results were shown in Figures 1A, 2A).

**Table 2:** Coordinate of significant regions in ANCOVA results.

**ANCOVA results**	**Regions**	**Coordinate (X Y Z)**	**Peak intensity**
Amygdala results	Right middle cingulate	9, 12, 36	10.7118
Hippocampus results	Bilateral medial superior frontal gyrus	0, 36, 45	9.5268

*Location of significant brain regions in MNI space*.

### Hippocampal rs-FC Results

The ANCOVA results showed that the bilateral medial superior frontal gyrus was the only area with a significant difference after correction (GRF correction, voxel *p* < 0.001, cluster *p* < 0.05, coordinate showed in Table 2). *Post hoc* analysis showed that compared with the other two groups, the FE-SZ group had a lower rs-FC between the hippocampus and the bilateral medial superior frontal gyrus (Bonferroni correction *p* < 0.05, also *p* < 0.001). There was no significant difference between the HR-SZ group and the HC group (*p* > 0.05) (the results were shown in Figures 1B, 2B).

### Correlation Analysis Results

**Figure 3:**
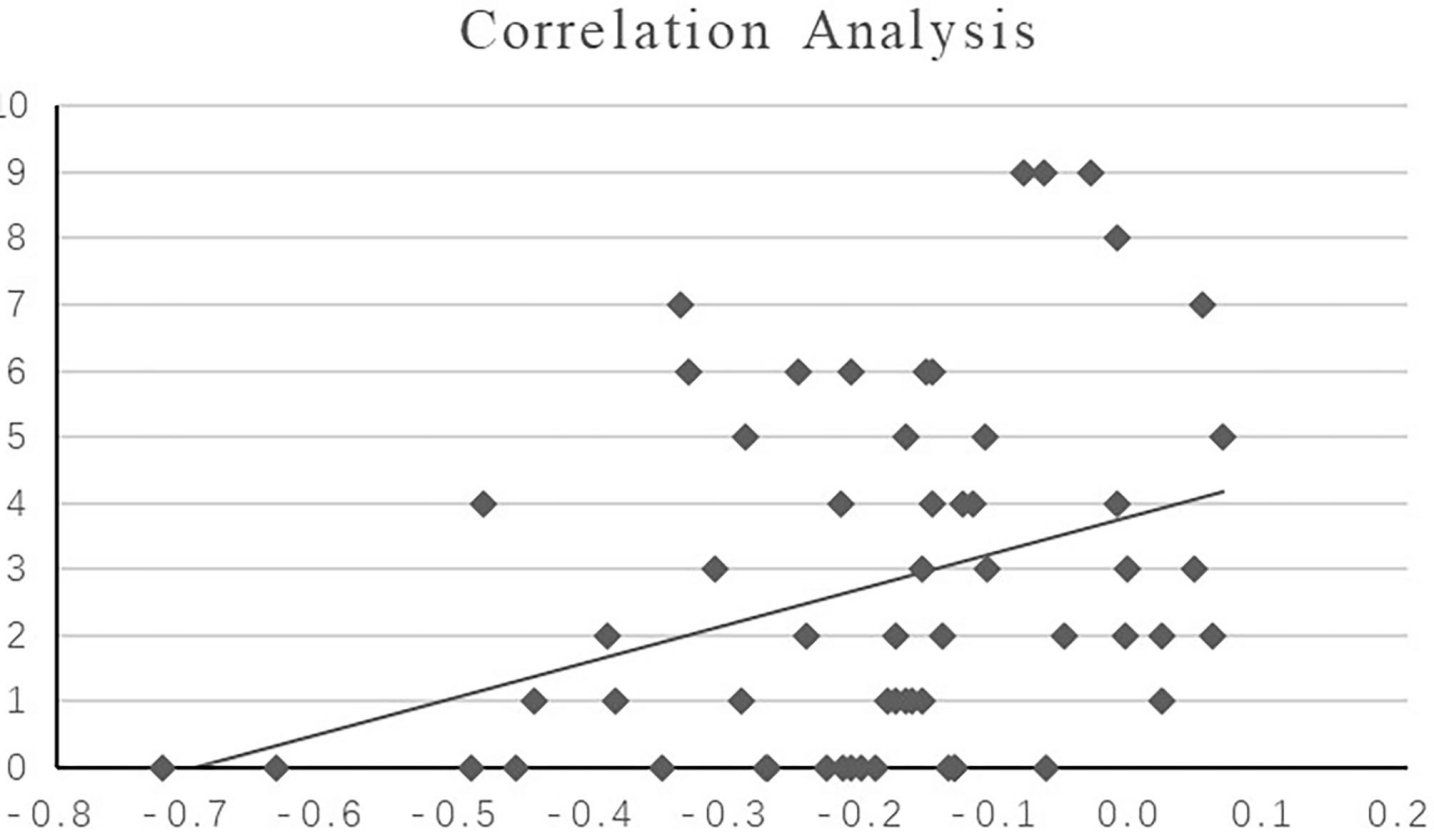
Correlation analysis between the HAMD core depression score and hippocampal-medial frontal lobe rs-FC value in FE-SZ. X-axis: the value of significant region (bilateral medial superior frontal gyrus); Y-axis: core depression score.

The hippocampal-medial frontal lobe rs-FC value in patients with FE-SZ was positively correlated with the HAMD core depression factor score (*P* = 0.004, *R* = 0.372, scatter plot was shown in Figure 3).

## Discussion

This study found that in FE-SZ, the rs-FC between the amygdala and the right middle cingulate and the rs-FC between the hippocampus and the bilateral medial frontal gyrus were reduced. There was no significant difference between HR-SZ and HC. Abnormal rs-FC in the FE-SZ was associated with depression symptoms. The results showed that there was no specific pattern in the high-risk population.

This study found a decrease in rs-FC in the amygdala-middle cingulate in the FE-SZ patients. The amygdala is an important component of the limbic system of the cortex and is thought to be involved in the early emotional processing of significant sensory stimuli (11, 26). The cingulate cortex is located on the inner surface of the cerebral hemisphere and it is an important part of the limbic system. It is involved in many important neural circuits and is closely related to the evaluation of emotional processing (27), such as the perception and interpretation of emotional stimuli (28). The cingulate cortex is divided into four functional regions: the anterior cingulate cortex, middle cingulate cortex, posterior cingulate cortex and posterior splenic cortex (29). The dorsal anterior cingulate cortex, commonly known as the middle cingulate cortex (30), is believed to be involved in cognitive processing, especially reward-based decision-making.

Our finding is consistent with the findings of previous research. In previous comparative studies of SZ patients and HCs, FC from the amygdala to the cingulate cortex and dorsolateral prefrontal cortex decreased significantly in emotional value (positive or negative), emotional consistency (same or different emotional content in the face and scene), and attention load tasks (10). The decrease in FC of the amygdala and middle cingulate may be related to the deficiency of SZ patients in processing significant emotional stimuli. It is difficult for affected patients to judge the importance of emotional stimuli and respond accordingly, which is manifested in clinical symptoms such as apathy and emotional incoordination. In the resting state, when the patient's positive symptoms are not obvious, the functional connection of the amygdala-middle cingulate may be used as a disease state imaging indicator of SZ.

Our study also found a decrease in rs-FC in the hippocampal-medial frontal gyrus (mPFC) in SZ patients. The mPFC is an important part of the prefrontal lobe and is the core area involved in advanced cognitive function. It is the center used for processing social information and memory functions related to the past and the center used for making long-term decisions for the future. Previous studies have found that the mPFC participates in the regulation of stress-related responses and participates in the regulation of emotions (31). Many cognitive and social neuroscience studies in healthy people have shown that the mPFC is one of the key areas for regulating emotional effects in understanding different texts and coherent judgment tasks (32, 33). In addition, in a study of schizophrenia, it was found that in the story understanding task of schizophrenia, there is a functional defect in the medial frontal gyrus area, which involves the interaction of several roles, indicating that the mPFC also plays a certain role in language comprehension (34). These results indicate that the lack of mPFC function in emotional processing in schizophrenia involves general emotional and cognitive aspects and is not limited to language or facial processing. Studies in animals and humans have shown that the hippocampus is similar to a network hub and uses information collected by tissues to activate specific cognitive patterns and to guide behaviors (35). In previous studies of patients with SZ, most of the tasks related to working memory in the task state found that the functional connection between the hippocampus and DLPFC was reduced. These studies have revealed the impact of impaired hippocampal-prefrontal functional connections on cognitive memory in SZ, but most of the studies were performed in a task state and did not reflect the brain function corresponding to some functional defects that exist in SZ in the resting state. This study found that the functional connection between the hippocampus and the mPFC region of SZ patients was reduced in the resting state. This is consistent with the results of a recent large study that included 509 Chinese Han SZ patients and found that compared with HC, the hippocampal volume and hippocampal-mPFC FC of patients were decreased compared with HC and compared with polygene assessment. The risk assessment score was negatively correlated, indicating that these two imaging changes are closely related to the pathophysiology of SZ (36). In addition, this study also found that the hippocampus-medial frontal lobe FC value in patients with FE-SZ was positively correlated with the HAMD core depression factor score (*P* = 0.006, *R* = 0.357). This is also in line with hippocampal-medial prefrontal dysfunction, which may reflect the emotional defects of SZ and may be a state imaging sign of SZ disease.

In this study, we did not find any changes in the functional connection between HR-SZ patients and HCs. Previous studies have not found any reports of abnormal resting-state functional connections in this functional loop of HR-SZ, most of which are studies on brain structure and task-state MRI. Keshavan et al. conducted structural MRI studies of 17 HR-SZ and 22 HC aged 13–22 years, compared intracranial volumes of DLPFC and hippocampal/amygdala complexes between the two groups, and found that the left side of HR-SZ of the hippocampus and amygdala complex decreased while the intracranial volume of DLPFC did not change (22). Sismanlar et al. conducted a study of HR-SZ and 22 HCs in 26 adolescents aged 8–15 and compared the intracranial volumes of the hippocampus, thalamus, corpus callosum, amygdala, frontal lobe, and temporal lobe of the two groups and found that the intracranial volume of the hippocampus and corpus callosum of SZ decreased significantly (37). In addition, in a task-state functional magnetic resonance study of 19 HR-SZ and 25 HC in a continuous emotional performance study, subjects continuously evaluated the emotional signals from a face in a particular trial (regardless of race identity), and compared with HC, it was found that HR-SZ had reduced activity of the left medial nucleus of the amygdala when faced with positive emotions. These results were not related to behavior/cognitive performance (equal among the groups), indicating that amygdala-impaired activation of emotional responses may be at the core of social dysfunction in this population (38). Additional similar research found that HR-SZ and HCs showed significantly different causal connection patterns between the amygdala and prefrontal cortex when shown faces with different emotional expressions (39). These results may support the hypothesis that emotional processing deficits may be a feature of an increased risk of schizophrenia (40). At the same time, other studies have found that the working capacity of the HR-SZ's anterior cingulate regulating the cortical system decreases during working memory tasks (41). However, research on the hippocampal connectivity of HR-SZ in our study circuit was limited. Combining the results of the above studies and the findings of this study, we can speculate that during the growth and development of a high-risk population, the volume of some areas of their cortical limbic system may be abnormal, which leads to the indicators changing on functional MRI in the face of certain emotions or cognitive tasks, but there may be no difference from HC in the resting state, which is reflected in their normal clinical, emotional, cognitive and social functions. Our results may effectively complement previous research findings.

This study has several limitations. Some patients with FE-SZ in this study were being treated with drugs, and the effects of these drugs on the functional connection results has not been fully elucidated. Smoking and drinking status might influence rs-FC in schizophrenia. However, in view of the integrity of the data, we did not consider the impact of smoking and drinking on the results.

In conclusion, there are different patterns of functional impairment in the amygdala and hippocampal neural circuits in the schizophrenic cortical-limbic system. Changes in the functional connections between the amygdala and the dorsal cingulate and between the hippocampus and the medial prefrontal lobe may be related to emotional symptoms and may be a state-related aberrance of the disease. This study did not find a clear amygdala/hippocampus functional connection change in currently unaffected offspring of SZ that may be related to genetics.

## Data Availability Statement

The data analyzed in this study is subject to the following licenses/restrictions: the datasets are not publicly available. Requests to access these datasets should be directed to Yanqing Tang, tangyanqing@cmu.edu.cn.

## Ethics Statement

The studies involving human participants were reviewed and approved by the study was approved by the Medical Science Research Ethics Committee of the First Affiliated Hospital of China Medical University (Approval Reference No. 2014[97]). The patients/participants provided their written informed consent to participate in this study.

## Author Contributions

XW designed the study, collected and analyzed the data, and drafted the manuscript. ZY collected and analyzed the data. XJ collected the data. QS and LC performed the MRI scan. XD and YT designed the study and revised the manuscript. All authors contributed to the article and approved the submitted version.

## Conflict of Interest

The authors declare that the research was conducted in the absence of any commercial or financial relationships that could be construed as a potential conflict of interest.

## Publisher's Note

All claims expressed in this article are solely those of the authors and do not necessarily represent those of their affiliated organizations, or those of the publisher, the editors and the reviewers. Any product that may be evaluated in this article, or claim that may be made by its manufacturer, is not guaranteed or endorsed by the publisher.
